# Effects of Cognitive Style and Evaluation Context on Hedonic and Sensory Perception of Café Latte: A Comparison of Sensory Booth, Real-Life, and Mixed Reality Environments

**DOI:** 10.3390/foods15091487

**Published:** 2026-04-24

**Authors:** Dongju Lee, Sangoh Kim, Seongju Woo, Youngseung Lee

**Affiliations:** 1 Department of Food Science and Nutrition, Dankook University, Cheonan-si 31116, Republic of Korea; dongju042027@naver.com; 2 Department of Food Engineering, Dankook University, Cheonan-si 31116, Republic of Korea; samkim@dankook.ac.kr (S.K.); usj0326@dankook.ac.kr (S.W.)

**Keywords:** coffee beverage, immersive technology, mixed reality, cognitive style, ideal CATA

## Abstract

This study examined cognitive style-related differences (analytic vs. holistic) in consumer liking, sensory perception, and ideal sensory profiles across three evaluation contexts (real café, sensory booth, and mixed reality). A total of 77 participants were divided into an analytic group (N = 34) and a holistic group (N = 43) based on the Analysis–Holism Scale. They evaluated six café latte samples varying in sugar concentration (0, 2.5, 5%) and espresso-to-milk ratio (1:2 and 1:3) for three environments using a within-subject design. Consumer evaluation comprised overall liking and sensory perception assessed using CATA (25 attributes) and Ideal CATA, with descriptive analysis (DA) conducted in parallel by eight trained panelists. The results showed no significant differences between cognitive styles in overall liking, but differences appeared in sensory perception and ideal product mapping between the booth and real café. The analytic group focused on dominant attributes with little variation for environments, whereas the holistic group integrated contextual cues, showing more context-dependent patterns. Compared with the other two environments, the MR environment showed high similarity to the DA results in terms of attribute profiles (RV = 0.88). This study indicates that cognitive style is a key factor in consumer sensory evaluation and should be considered to improve sensory evaluation methodology.

## 1. Introduction

The development and launch of new products in the food and beverage industry are considered key strategies for securing corporate competitiveness and ensuring sustainable growth [1]. However, despite various efforts to predict consumer acceptance prior to product launches, a high rate of product failure continues to be reported in the food industry [2]. These failures result from the combined influence of multiple factors and raise questions regarding whether conventional sensory evaluation can accurately predict actual consumer preferences, choices, and purchasing behavior [1]. Consumer preferences and choices are shaped not only by biological, psychological, and sociocultural factors but also by intrinsic and extrinsic product characteristics and the contextual environment in which the product is consumed [3].

Traditional sensory evaluation is typically carried out in controlled environments such as sensory booths or central location tests, where external stimuli such as lighting, color, aroma, and noise are strictly controlled, and partitions or individual booths are used to prevent interaction among consumers [4,5]. While this approach standardizes contextual cues to ensure that evaluation results reflect the intrinsic characteristics of the product, it may reduce consumer immersion and ecological validity, potentially distorting sensory responses [6,7]. To overcome these limitations, home-use tests, which reflect real consumption environments, have been employed as an effective alternative for enhancing the external validity of sensory evaluation. Data collected in real contexts contribute to more accurate predictions of food choice and increase the practical applicability of evaluation results [8]. However, sensory evaluation in natural environments is limited by reduced experimental control, difficulties in standardizing test conditions, and higher time and cost requirements [1,8,9].

Therefore, as an alternative to simultaneously ensuring internal and external validity, the use of immersive environments for sensory evaluation has recently gained attention in both industry and academia [2]. Immersive environments provide a sense of realism through virtually simulated or physically reproduced consumption contexts, while maintaining a high level of experimental control, and are thus regarded as a novel approach in sensory evaluation [4]. Such environments are implemented using multisensory cues (e.g., visual, auditory, olfactory, and tactile stimuli) and can be created using props, images, scenario-based stimuli, screens, or extended reality (XR) technologies [10]. XR encompasses virtual reality (VR), augmented reality (AR), and mixed reality (MR), which differ in the level of immersion and the mode of interaction with the real environment. VR provides a fully virtual environment, AR overlays digital information onto the real space, and MR enables real-time interaction and coexistence between real and virtual elements [11]. Notably, MR has been reported to provide higher immersion, more intuitive interaction, and greater contextual coherence than AR and VR, and has been suggested as a valuable alternative for improving both the internal and external validity of sensory evaluation [12].

Traditional sensory evaluation also has the limitation of insufficiently reflecting psychological variables that influence consumer perception and response. Among these, cognitive style has been highlighted as an important psychological factor affecting how consumers perceive and evaluate products, and its importance has been increasingly emphasized in recent sensory and consumer research [13]. In particular, the analytic–holistic (AH) cognitive theory is commonly linked to cultural differences between Western and Eastern societies. Analytic thinking, often observed in Western cultures, is associated with object-focused attention, linear reasoning, and independent interpretation of events, whereas holistic thinking, typically observed in Eastern cultures, is associated with context-dependent attention, cyclical interpretation and prediction, and recognition of interdependent relationships between events [14].

Differences in thinking styles and contextual factors arising from cultural background thus lead to differences in sensory perception and preference, meaning that consumers may perceive and evaluate the same product in different ways. Accordingly, product development requires optimization strategies that reflect the expectations and needs of diverse consumer groups. In particular, consumer-oriented optimization requires sensory approaches capable of quantitatively identifying ideal product characteristics, and methods such as Ideal check-all-that-apply (CATA) serve as useful tools for deriving ideal points and visualizing product attributes [15].

To overcome the limitations of conventional sensory evaluation environments, numerous studies have been conducted using immersive methods. Ref. [2] performed consumer segmentation based on sensory acceptance tests with beer samples conducted in sensory laboratories, immersive environments, and real consumption settings, and analyzed the influence of contextual environments on consumer responses. Ref. [16] investigated the effect of contextual elements on participant experiences using MR with wine samples, virtual questionnaires, and custom-made wine glasses in sensory booth and wine bar settings. In addition, coffee beverages have frequently been used as test samples in sensory evaluation studies conducted in immersive environments [17]. Coffee is a representative beverage that is frequently consumed in socially rich environments such as cafés, where atmospheric cues including lighting, background music, and spatial ambience may influence consumer perception and evaluation [18].

Although many studies have examined the application of immersive environments in sensory evaluation, there remains a lack of research comparing product optimization across sensory booths, real consumption environments, and immersive settings while considering cognitive style as a moderating factor. In practice, although consumers’ ideal sensory profiles may vary across consumption contexts, sensory booths are still predominantly used in practice due to their high control and reproducibility, which may limit the extent to which real consumption contexts are adequately captured. Therefore, the primary objective of this study was to examine cognitive style-related differences in consumer liking, sensory perception, and ideal profiles across three evaluation contexts; additionally, we evaluated context-related variations in consumer response patterns in analyses that did not stratify participants by cognitive style.

## 2. Materials and Methods

### 2.1. Sample

Six types of café latte were prepared as samples by varying the sucrose content and the ratio of milk to espresso (Table 1). All coffee capsules (Nespresso, Nestlé Nespresso SA, Lausanne, Switzerland) were purchased from the official Nespresso online store, while white sugar (Baeksul, CJ Cheiljedang Corp., Seoul, Republic of Korea) and milk (Seoul Milk, Seoul Dairy Cooperative, Seoul, Republic of Korea) were obtained from a local supermarket.

According to Spence [18], consumers’ sensory perception of coffee is greatly influenced by contextual factors such as lighting, spatial atmosphere, and background music. Considering this, café latte was selected as the test beverage, as it is suitable for examining perceptual differences across evaluation environments and for eliciting a broad range of sensory attributes. To ensure product consistency and minimize external variability related to equipment, coffee beans, and operator skill, a Nespresso coffee machine (Essenza Mini C30, black; Nestlé Nespresso SA, Lausanne, Switzerland) was used with the Ispirazione Italiana Venezia capsule. Each capsule contained approximately 5 g of ground coffee, as specified by the manufacturer. Espresso was extracted according to the manufacturer’s standard protocol, yielding approximately 40 mL of beverage per capsule. Based on the coffee dose and beverage yield, the extraction corresponded to a brew ratio of approximately 1:7 (coffee:water), which falls within the range commonly reported for capsule-based espresso preparation systems. The extraction parameters followed the standard operating specifications of the Nespresso Essenza Mini C30 system. According to the manufacturer, the brew water temperature during extraction is approximately 83–86 °C and the pump pressure reaches up to 19 bar. The extraction time for a standard espresso capsule is approximately 25 s. Because the capsule system is fully automated, these extraction parameters (temperature, pressure, flow rate, and extraction time) were maintained constant across all sample preparations. This capsule is produced from South American beans subjected to medium-dark roasting at a medium intensity (intensity: 8), characterized by a balanced profile of moderate bitterness and acidity with notes of roasted aroma and caramel. Moreover, it is known to retain a harmonious flavor when blended with milk and sugar.

For sample preparation, milk for all samples was heated and frothed using the same milk frother (Aeroccino3, Nestlé Nespresso SA, Lausanne, Switzerland) under identical frothing conditions, and the frothed milk was subsequently mixed with extracted espresso at coffee-to-milk ratios of 1:2 and 1:3. The same frothing conditions were applied for both milk ratios. The 1:2 ratio was selected as the standard latte proportion recommended by Nespresso, while the 1:3 ratio was included to reflect the attenuation of coffee flavor with increased milk content and to enhance the discriminability among samples. Sucrose was added at concentrations of 0, 2.5, and 5% of the final beverage weight, based on preliminary trials and previous reports indicating that the maximum sugar concentration commonly used in beverages is approximately 10% [19]. At 10%, excessive sweetness masked the intrinsic coffee flavor, whereas concentrations below 5% maintained an appropriate balance between sweetness and coffee aroma. These concentration levels were further validated by a trained descriptive panel, confirming that they provided sufficient sensory discrimination among samples. All samples were stored in an insulated flask immediately after preparation to minimize temperature changes and were evaluated within one hour of production. Each sample was labeled with a randomly assigned three-digit code generated from a random number table and served in identical 380 mL paper-based take-out cups containing 100 mL of beverage at 55 ± 2 °C, together with a resealable lid and a cup holder. All samples were freshly prepared following the same procedure prior to each evaluation session.

### 2.2. Descriptive Analysis

A trained panel consisting of eight adults in their twenties (one male and seven females) who demonstrated high accuracy in screening tests for basic tastes, flavors, and textures was selected. This age group was selected because sensory acuity is generally higher in younger adults and tends to decline with age [20]. The selection criteria considered sensory acuity, the objectivity and diversity of sensory expression, the absence of food allergies, and the ability to participate in long-term evaluations [21]. Prior to the main training, the selected panelists received instruction on the concepts of the evaluation procedure and the use of the intensity scale. Training was conducted twice weekly for three months, with a total training time of approximately 16 h per month. Repeated exercises were included to ensure consistency and reproducibility of the evaluations. During the training period, repeated rating exercises using reference samples and a 16-point numerical intensity scale were conducted to improve both inter- and intra-panel agreement and ensure reproducibility. Prior to the formal evaluations, panel performance was monitored during training sessions to ensure consistency in attribute recognition and scale usage. Panelists demonstrating inconsistent responses were provided with additional feedback and practice using reference standards to improve agreement.

After the basic training, the panel reviewed sensory attributes of café latte reported in the literature and then evaluated café latte samples. During these sessions, panelists were encouraged to freely propose additional sensory terms. The resulting list was refined through group discussion to remove redundant or ambiguous descriptors, and only terms unanimously agreed upon by all panelists were retained as the final descriptor list and sensory lexicon. In parallel, reference standards were selected for each attribute, and the intensity levels of these standards were adjusted through iterative, consensus-based discussions until a shared set of intensity anchors was established. Through this process, a sensory lexicon comprising 25 attributes covering aroma, flavor, and texture was established (Table 2). Sample evaluations were carried out in individual booths maintained at 21 ± 2 °C, following a completely randomized block design, with each panelist evaluating each sample twice. Samples were evaluated in two sessions of three samples each, with a 10 min break between sessions. Each sample was served in 100 mL portions at 55 ± 2 °C. The trained panelists evaluated each attribute on a 16-point intensity scale ranging from 0 (none) to 15 (very strong). Panelists rinsed their mouths with water before and after each evaluation. The entire evaluation procedure was replicated twice on separate days.

### 2.3. Consumer Test

#### 2.3.1. Participants

Participants in this study were recruited from university faculty, staff, and students through a student-exclusive social networking platform and on-campus recruitment notices. Because the same participants were required to complete consumer evaluations under all three conditions (real café, sensory booth, and MR), university members were selected in consideration of accessibility and sustained participation. Individuals younger than 20 or older than 60 years, those with difficulties consuming liquid foods, and those with milk or caffeine allergies or lactose intolerance were excluded.

As a result, 77 healthy adults aged 20–60 years participated in the study (mean age: 26.0 ± 5.1 years), including 55 females and 22 males. This number of participants is consistent with commonly used sample sizes in consumer sensory evaluation studies [22]. The study protocol was approved by the Institutional Review Board of Dankook University (DKU 2025-01-025-002), and all procedures were conducted in accordance with the committee’s guidelines.

#### 2.3.2. Assessment of Analytic–Holistic Cognitive Style

Prior to participation in the evaluation, all participants completed the Analytic–Holistic Scale (AHS) developed by Choi [23] as part of a preliminary screening procedure to quantitatively assess their AH tendency. The AHS consists of 24 items, each rated on a 7-point Likert scale ranging from 1 (strongly disagree) to 7 (strongly agree) [13]. The scale is structured around four core factors: locus of attention, causal theory, perception of change, and attitude toward contradictions.

According to Choi [23], the AHS demonstrated an internal consistency of Cronbach’s α = 0.74. In the present sample, the internal consistency of the AHS was modest (Cronbach’s α = 0.66) and falls within the range reported in previous AHS studies, typically around 0.60–0.75 [24,25]. Higher mean scores across the 24 items indicate a stronger holistic tendency, whereas lower mean scores reflect a stronger analytic tendency. In previous studies that quantitatively assessed AH tendencies [14,26,27], classification of participants into analytic or holistic groups was based on criteria such as mean values, median values, or the upper and lower 27% of the distribution. Among these, Beekman [14] classified participants into holistic and analytic groups through within-culture comparisons, reporting mean values of 5.46 for the holistic group and 4.63 for the analytic group. In the present study, the median of the overall AHS mean scores was 4.83. Participants with AHS scores below this median were classified into the analytic group, whereas those with scores above the median were classified into the holistic group, in order to minimize the influence of extreme values. As a result, the analytic (N = 34) and holistic (N = 43) groups had mean AHS scores of 4.56 and 5.33, respectively, which were consistent with the trends reported in previous research [14]. However, because both groups in the present study fall within a moderately to highly holistic range, the contrast between the “analytic” and “holistic” groups should be interpreted as a relative rather than an absolute distinction.

#### 2.3.3. Experimental Design

This study employed the AHS to classify participants according to their AH tendency and subsequently conducted a consumer study based on a 3 × 6 full factorial design, with evaluation environment (real café vs. sensory booth vs. MR) and product type (six café latte samples) as factors. When comparisons across environments are conducted using a between-subjects design, even if groups are matched based on observable variables, there remains a limitation in sufficiently accounting for differences inherent to the environments themselves [2]. Therefore, a within-subjects design was applied, in which the same participants evaluated all environmental conditions, to enable a more accurate analysis of environmental effects. All participants completed the evaluations in a fixed order: real café, sensory booth, and finally MR.

The test for sensory booth condition was conducted in individual partitioned booths within the Sensory Laboratory at Dankook University. Each booth measured 80 cm in width and 100 cm in depth and was equipped with a desk for sample placement and questionnaire completion. The booths were designed to control for non-product-related influences such as external odors, lighting, and noise. White LED lighting was used, with horizontal illuminance at the work surface maintained at approximately 400–500 lux, and background noise kept below 40 dBA. The room temperature was maintained at 21 ± 2 °C. The real café condition was conducted at Yogerpresso, a commercial café located near Dankook University, which served as the real café setting in this study. Participants were seated at tables within the café and carried out tasting under conditions consistent with a natural consumption environment, including the presence of background classical music. During the evaluation sessions, the average illuminance at the table surface was approximately 100–200 lux, the background noise level was around 60–70 dBA, and the indoor temperature was maintained at approximately 24 ± 2 °C. The test for the MR condition was conducted using an MR headset (Meta Quest 3S, Meta Platforms Inc., Menlo Park, CA, USA), through which participants experienced an immersive environment replicating the spatial features of the actual café.

In this study, MR technology was applied to reproduce the experience of drinking coffee in a real café environment within a virtual space. The interior of the selected café was recorded as a 10 min 360° video using an Insta360 X4 camera (Insta360; Arashi Vision Inc., Shenzhen, Guangdong, China). The camera was mounted on a tripod at eye level when seated, and its position was adjusted to allow participants to feel as if they were sitting at a café table. The recorded video was edited using Insta360 Studio software (version 5.5.3; Insta360; Arashi Vision Inc., Shenzhen, Guangdong, China) at a resolution of 4096 × 2048 and a frame rate of 29.97 fps. The edited video was then implemented into an MR headset through the Unity 6 program (Version 6000.0.32f1; Unity Technologies, San Francisco, CA, USA). To allow participants to view the actual questionnaire and samples within the virtual environment, the Passthrough function of the Meta Quest 3S was activated, and a window was embedded into the virtual space. The position of the virtual café table was precisely aligned with the real table so that participants perceived the table as physically present within the virtual environment. For auditory immersion, the actual background sounds of the café, recorded during filming, were played during the evaluation through a Bluetooth speaker (Bose SoundLink Mini II; Bose Corporation, Framingham, MA, USA).

#### 2.3.4. Sensory Procedure

Prior to participation, all subjects were informed of the study purpose, procedures, and data protection policies, and written consent for the use of personal information and research participation was obtained. Participants then evaluated six café latte samples presented in a randomized order according to a William’s Latin Square design. The samples were assessed sequentially using an overall liking scale, a CATA questionnaire, and an Ideal CATA task. A small gift was provided to participants upon completion of the study.

The acceptance test was conducted using a 9-point hedonic scale ranging from 1 (dislike extremely) to 9 (like extremely) to assess overall liking. Liking scores were further categorized into “dislike” (1–4) and “like” (5–9). Following the liking assessment, participants completed a CATA questionnaire, which was developed based on the sensory lexicon established by the DA panel. The questionnaire included 25 sensory terms relevant to café latte, classified into aroma (coffee, chocolate, nutty, caramelized, roasted, vanilla, sweet, and burnt), taste/flavor (sweet, sour, bitter, umami, burnt, coffee, caramelized, nutty, chocolate, milk, creamy, and aftertaste), and texture (smooth swallowing, mouth coating, residual, chalky coating, and astringency) categories (Table 2). In the CATA task, participants were instructed to select all terms they considered appropriate to describe each sample. Finally, after evaluating all samples, participants completed an Ideal CATA task using the same questionnaire. They were asked to indicate the attributes perceived in their “ideal” café latte by responding to the question: “Please select all characteristics that you consider to be present in your ideal café latte.”

**Figure 1:**
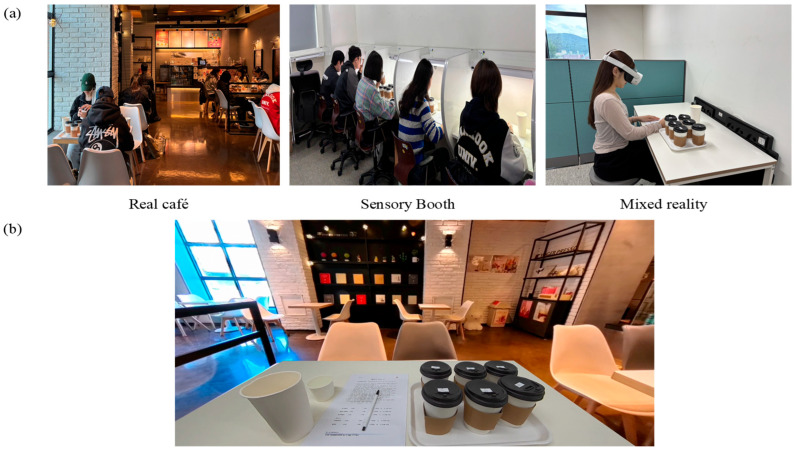
Experimental settings for the sensory evaluation of café latte sample. (**a**) Test environments for three consumer sensory evaluation conditions, (**b**) Representation of participants’ view in the MR condition.

For the MR condition, the following additional procedure was applied to ensure participants’ adaptation to the headset and the virtual environment. The evaluation procedure required participants to wear the MR headset and first spent approximately one minute adapting to the device and visual field, followed by an additional minute of immersion in the virtual café environment while drinking water. After sufficient adaptation to both the equipment and the environment, participants proceeded to taste the café latte samples and complete the questionnaire through the Passthrough window. During the tasting, participants were able to see their own hands and the real cup as they brought the beverage to their mouths, thereby maintaining natural drinking movements. Video 1 illustrates participants’ view in the MR condition, and the conditions of all evaluation environments are visually presented in Figure 1.

### 2.4. Statistical Analysis

DA data were analyzed using analysis of variance (ANOVA), with samples as fixed factors and panelists as random factors. When significant differences among samples were identified, Tukey’s honest significant difference (HSD) test was conducted as a post hoc analysis. Principal component analysis (PCA) was conducted to visualize the multivariate relationships between samples and sensory attributes in a reduced-dimensional space, including only attributes that showed significant differences in the ANOVA.

Consumer acceptance data were analyzed using one-way repeated-measures ANOVA, with context (evaluation environment) as the fixed factor and participant as the random factor. Within each evaluation environment (real café, sensory booth, MR café), differences among the six latte samples were examined with the sample as the fixed factor. In a separate analyses, differences in overall liking for the three evaluation environments for each sample were examined using one-way repeated-measures ANOVA with environment (real café, sensory booth, MR café) as the fixed factor. Post hoc comparisons were conducted using Tukey’s HSD test at a significance level of 5% (*p* < 0.05). The effect size for the main effect of testing context on overall liking was small (partial eta-squared, ηp^2^ = 0.036). Differences in consumer responses were analyzed using mixed-effects models, with sample, context (evaluation environment), and cognitive style (analytic vs. holistic) as fixed effects. Participant was included as a random effect to account for repeated measures. Interactions among the fixed effects were also examined.

CATA results were analyzed using Cochran’s Q test to determine attribute-based differences among samples. The relationships between samples and attributes, including the ideal product, were visualized through correspondence analysis (CA). Penalty analysis was conducted based on overall liking and ideal product attributes. Attributes selected by more than 20% of consumers and associated with a mean drop greater than 1 point in overall liking were considered significant [28,29]. Multiple discriminant analysis (MDA) was also conducted to complement the correspondence analysis (CA) results. The agreement among different analytical outcomes was evaluated using the RV coefficient, with values ≥ 0.7 considered acceptable [30]. To account for multiple testing, *p*-values from the Cochran’s Q tests for the 25 CATA attributes and from the RV coefficient comparisons were adjusted using the Benjamini–Hochberg false discovery rate (FDR) procedure [31]. For these analyses, statistical significance was determined based on FDR-adjusted *p*-values (q), with q < 0.05 considered statistically significant; values of 0.05 ≤ q < 0.10 were considered suggestive and interpreted with caution [32].

All statistical analyses were conducted using XLSTAT software (Version 2024, Addinsoft Inc., Paris, France), with a significance level set at *p* < 0.05.

## 3. Results

### 3.1. Descriptive Analysis

In the DA, a total of 25 sensory attributes were evaluated, and the mean intensities and ANOVA results for each sample are presented in Table 3. Significant differences among samples (*p* < 0.05) were observed for all sensory attributes except chocolate aroma, nutty aroma, umami, chocolate flavor, nutty flavor, aftertaste, and chalky coating. These attributes generally exhibit relatively low perceptual intensity or subtle differences in coffee beverages and may therefore be difficult to perceive consistently even by trained panelists. Nevertheless, significant differences were observed among samples for most sensory attributes, indicating that the samples were clearly discriminated across a wide range of sensory characteristics.

**Figure 2:**
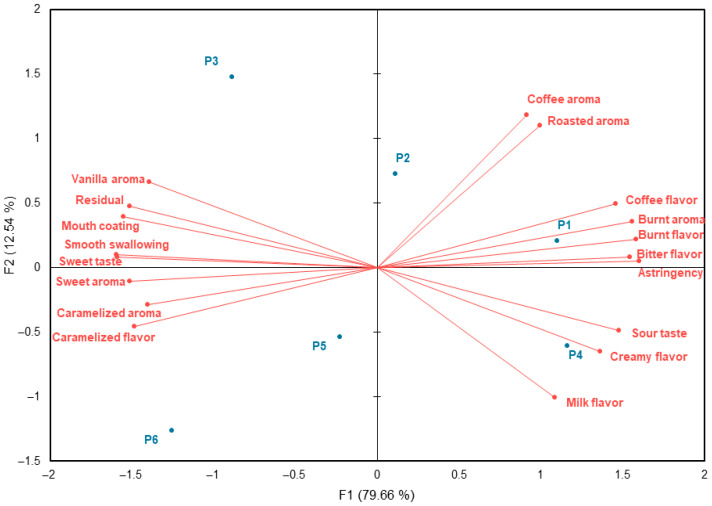
Principal component analysis (PCA) using significant descriptive sensory attributes.

PCA conducted using only the attributes that showed significant differences in the ANOVA, revealed that F1 (79.66%) and F2 (12.54%) together explained 92.2% of the total variance, clearly distinguishing sensory differences among samples (Figure 2).

F1 differentiated the samples according to sucrose concentration: sugar-free samples P1 and P4 were positioned on the positive axis of F1, whereas 5% sucrose samples P3 and P6 were located on the negative axis. P1 and P4 were generally characterized by pronounced coffee flavor and milk flavor. Specifically, P1 was associated with coffee aroma, burnt aroma, burnt flavor, bitter taste, and astringency, while P4 was characterized by sour taste, creamy flavor, and milk flavor. In contrast, P3 and P6 were strongly associated with sweetness-related attributes. P3 was characterized by vanilla aroma, residual, smooth swallowing, and sweet taste, whereas P6 exhibited strong associations with sweet aroma, caramelized aroma, and caramelized flavor. These results indicate that higher sucrose concentrations enhanced sweet taste, caramelized flavor, and smooth swallowing, whereas sugar-free samples emphasized coffee flavor with distinct burnt aroma, sour taste, and bitter taste.

Although F2 accounted for a smaller proportion of variance, it differentiated samples according to the espresso-to-milk ratio, particularly contributing to the separation between P2 and P5. Overall, within the set of café latte samples used in this study, sucrose concentration and the espresso-to-milk ratio appeared to be the main drivers of the sensory differences observed among samples.

### 3.2. Consumer Test

#### 3.2.1. Impact of Evaluation Environment and Cognitive Style on Overall Liking

Table 4 presents the ANOVA results for the effects of evaluation context, cognitive style, and their interaction on the overall liking of café latte samples. Across samples, no significant differences in overall liking were observed between the analytic and holistic groups. Overall, sucrose-added samples (P2, P3, P5, P6) received significantly higher liking scores than the sugar-free samples (P1, P4), indicating a substantial contribution of sucrose to product liking. In addition, significant differences between the two sugar-free samples (P1 vs. P4) were observed for both cognitive style groups in the real café and MR contexts, whereas under the sensory booth context this difference was not observed for the analytic group.

Table 5 summarizes the ANOVA results for overall liking of café latte samples across the three evaluation contexts. Consumers rated samples P2, P3, and P5 significantly higher in the real café than in the sensory booth. Furthermore, only P2 was rated significantly higher in the real café than in the MR context. Overall, sucrose-added samples (P2, P3, P5, P6) received higher liking scores than the sugar-free samples (P1, P4). Within the real café context, a significant difference in liking was observed between the two sugar-free samples (P1 vs. P4). In contrast, under the MR context significant differences were observed among P1, P2, and P4, whereas no such differences were observed in the sensory booth context.

#### 3.2.2. CATA-Based Sensory Attribute Analysis

Table 6 presents the results of the nonparametric Cochran’s Q test, showing significant differences in the frequency of sensory attributes among samples. In the real café environment, significant differences were observed for 16 attributes, excluding coffee aroma, chocolate aroma, nutty aroma, caramelized aroma, vanilla aroma, milk flavor, aftertaste, mouth coating, and residual. In the sensory booth, significant differences were identified for 13 attributes, excluding coffee aroma, chocolate aroma, nutty aroma, roasted aroma, vanilla aroma, umami, coffee flavor, milk flavor, creamy flavor, aftertaste, mouth coating, and residual. In the MR environment, significant differences were observed for 17 attributes, excluding coffee aroma, chocolate aroma, nutty aroma, roasted aroma, coffee flavor, nutty flavor, aftertaste, and residual.

**Figure 3:**
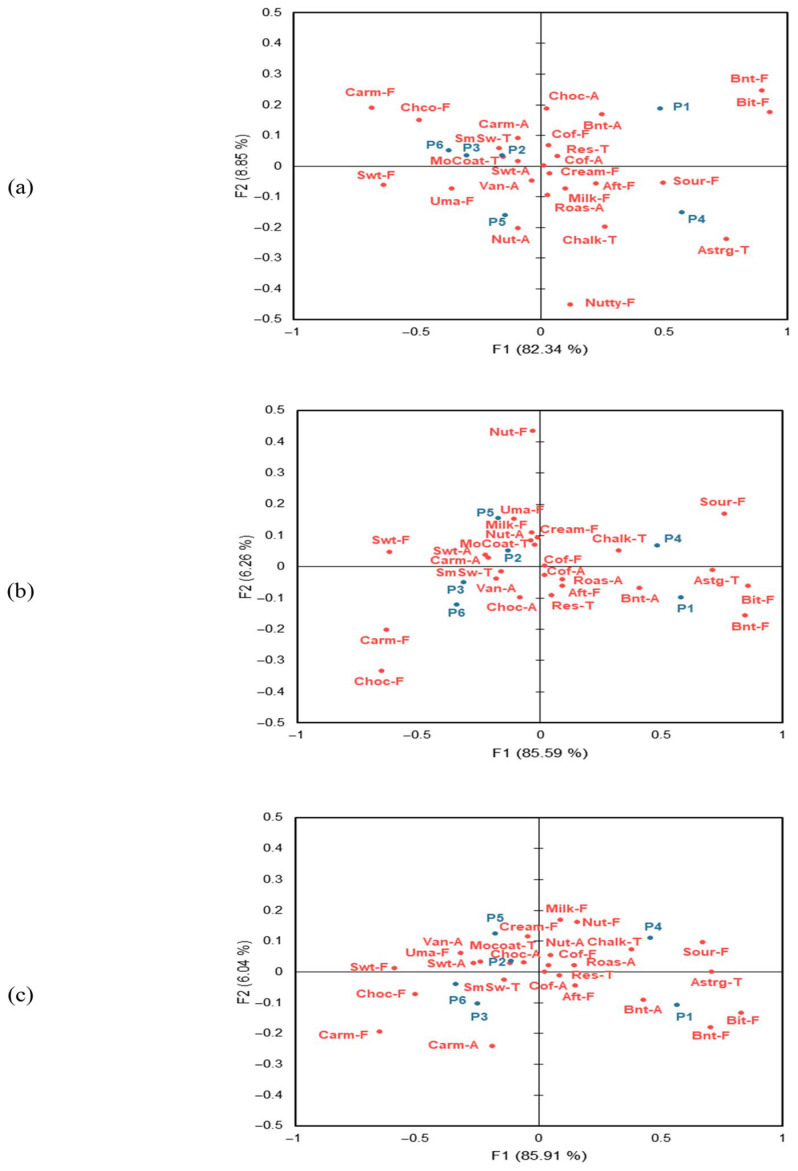
Correspondence analysis (CA) plot using the significant CATA attributes for different sensory evaluation environments. Sensory attribute abbreviations are defined in Table 2. (**a**): real café, (**b**): sensory booth, (**c**): mixed reality.

Figure 3 visualizes the relationships between samples and sensory attributes in the three evaluation environments based on CA. In the real café environment, F1 and F2 explained 91.19% of the total variance, while in the sensory booth and MR environments, they explained 91.85% and 91.95%, respectively, indicating that two dimensions accounted for most of the variability across all conditions (Figure 3a). For the three evaluation environments, F1 primarily reflected the relatively large step differences in sucrose concentration, yielding a consistent configuration of samples across conditions, whereas F2 was interpreted as the axis capturing subtle context-related differences in flavor and textural attributes. In the real café, P2, P3, and P6 were positioned in the positive direction of F2 and were strongly associated with caramelized aroma, smooth swallowing, and mouth coating, whereas P5 was located in the negative direction of F2 and closely related to sweet aroma, vanilla aroma, umami, and nutty aroma. In contrast, in both the sensory booth and MR environments, samples were clearly distributed according to sucrose concentration (Figure 3b,c). In both environments, P2 and P5 were located in the positive direction of F2, associated with sweet taste, sweet aroma, and mouth coating, while P3 and P6 were positioned in the negative direction of F2, strongly related to caramelized flavor and smooth swallowing. The sugar-free samples, P1 and P4, were distinctly separated from the other samples across all three environments, with P1 closely associated with burnt aroma, burnt flavor, and bitter taste, and P4 with sour taste and chalky coating. These findings suggest that, unlike in the real café, sensory booth and MR environments showed clearer separation by sucrose concentration.

To complement the CA results and quantitatively assess the relationships between samples and sensory attributes, MDA was conducted (Table 7). The cosine values ranged from −1 to +1, with absolute values ≥ 0.707 indicating strong associations between samples and attributes [22]. Across all environments, sour taste, bitter taste, burnt flavor, and astringency were strongly associated with P1 and P4. In the real café environment, P2, P3, and P6 showed strong associations with smooth swallowing. In contrast, in the sensory booth and MR environments, P3 and P6 were strongly related to caramelized flavor, chocolate flavor, and smooth swallowing. These results were consistent with the CA findings, providing quantitative confirmation of the relationships between samples and sensory attributes.

**Figure 4:**
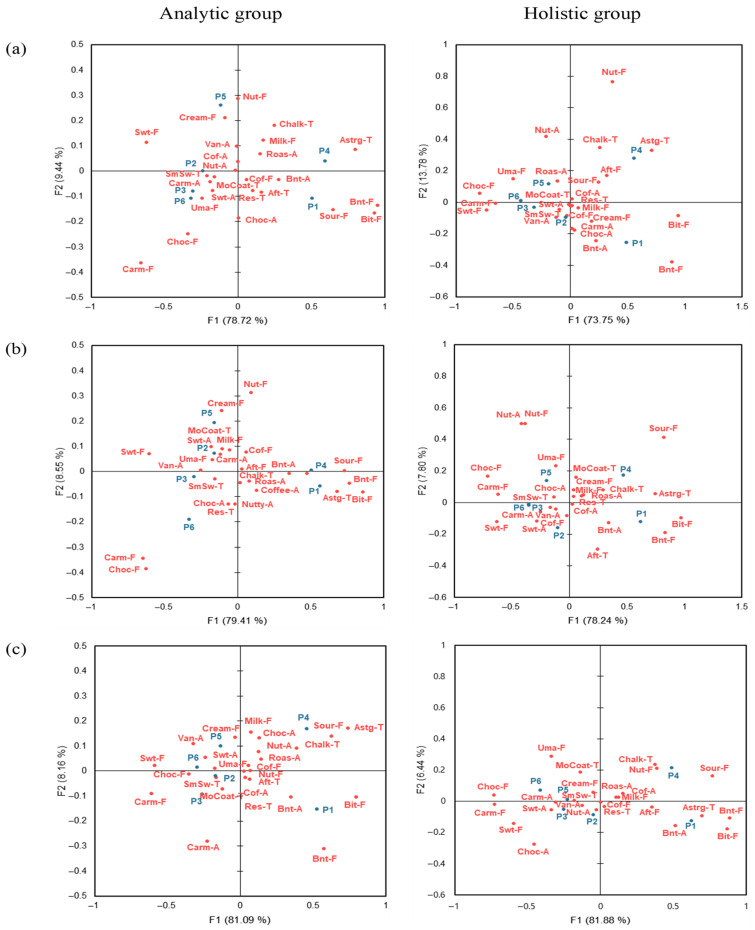
Correspondence analysis (CA) plot using the significant CATA characteristics for different sensory evaluation environments and cognitive styles. Sensory attribute abbreviations are defined in Table 2. (**a**): real café, (**b**): sensory booth, (**c**): mixed reality.

To address the primary objective, Figure 4 compares CA configurations between analytic and holistic consumers within each evaluation context. Because F1 primarily reflected the relatively large step differences in sucrose concentration and consequently yielded a largely similar configuration of samples across contexts, it was of limited use for discriminating cross-context differences; therefore, cross-context differences were mainly interpreted along F2. In the analytic group, samples with the same sucrose concentration (P2 and P5, P3 and P6) were located in the same quadrant (Figure 4a,b), indicating that samples with identical sucrose levels tended to cluster in a similar region of the CA space across contexts. P2 and P5 were associated with creamy flavor and vanilla aroma, while P3 and P6 were strongly related to smooth swallowing. In the holistic group, the real café samples were separated along F2 according to the espresso-to-milk ratio, indicating that holistic processors were more sensitive to the global beverage composition in this context: P5 and P6 were associated with umami and chocolate flavor, whereas P2 and P3 were linked to sweet aroma, smooth swallowing, and vanilla aroma (Figure 4a). In the sensory booth, P2, P3, and P6 were closely associated with caramelized aroma, vanilla aroma, coffee flavor, sweet aroma, and sweet taste, indicating that the holistic group exhibited context-dependent variations in sample distribution (Figure 4b). In the MR environment, samples in both cognitive style groups tended to be separated along F2 according to the espresso-to-milk ratio. P5 and P6 were related to sweet aroma, umami, creamy flavor, and vanilla aroma, whereas P2 and P3 were strongly associated with chocolate flavor, smooth swallowing, and mouth coating (Figure 4c). The sugar-free samples, P1 and P4, were consistently located in separate quadrants from sucrose-added samples across all environments and cognitive styles, with P1 strongly associated with burnt flavor, burnt aroma, and bitter taste, and P4 with chalky coating. Although this overall separation between sugar-free and sucrose-added samples was stable across conditions, the spatial arrangement of each sample and its associations with specific sensory attributes differed according to evaluation context and cognitive style (Figure 4).

#### 3.2.3. Penalty Analysis

Penalty analysis categorizes attributes into must have, nice to have, do not harm, and must not have, based on the impact of differences in attribute selection frequency between actual and ideal products on consumer liking [33]. Among these, must have attributes refer to characteristics that must be present in a product to secure consumer acceptance, as their absence leads to a significant decrease in liking. Conversely, the must not have attributes are those absent in the ideal product but, when present in the actual product, decrease consumer liking and thus should be excluded.

**Figure 5:**
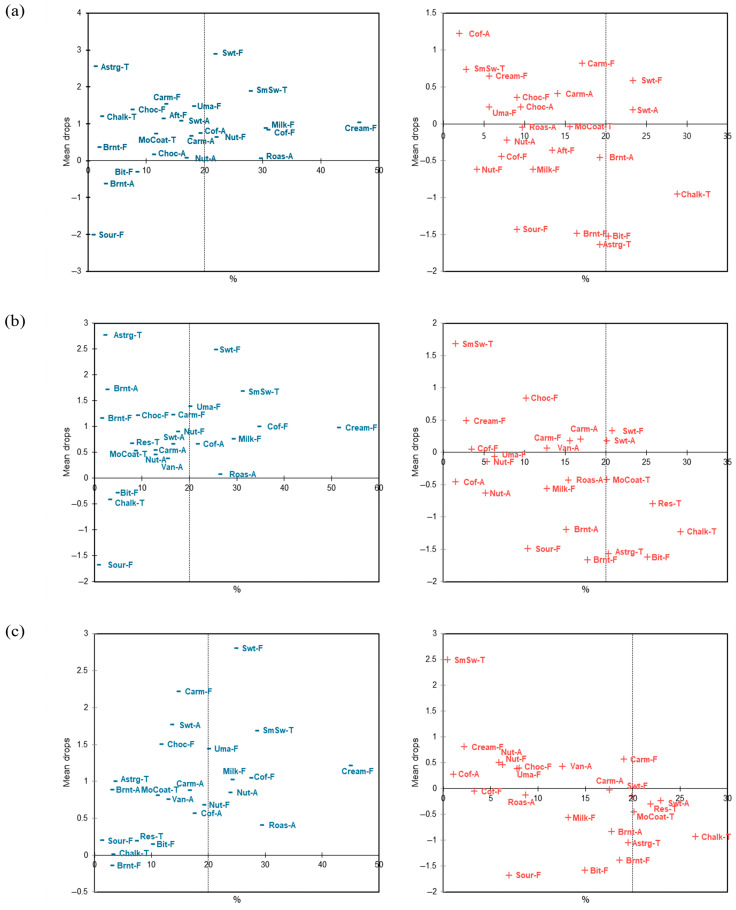
Penalty analysis plot for café latte samples according to different sensory evaluation environments. ‘Must have’ attributes are shown in blue, whereas ‘Nice to have’ or ‘Must not have’ (attributes not selected for the ideal product but observed in the real samples) are shown in red. Sensory attribute abbreviations are defined in Table 2. (**a**): real café, (**b**): sensory booth, (**c**): mixed reality.

As a secondary, exploratory analysis, penalty analysis was conducted within each evaluation context without stratifying by cognitive style, and the attributes were classified and visualized as must-have, nice-to-have, and must-not-have categories (Figure 5). Following the criteria commonly used in previous penalty-analysis studies [28,29], among the attributes selected by at least 20% of consumers, those with mean drop values ≥ 1 were defined as must have, whereas those with mean drop values ≤ −1 were defined as must not have. In the real café environment, sweet taste, smooth swallowing, and creamy flavor were identified as must have attributes (Figure 5a). In the sensory booth, sweet taste, smooth swallowing, creamy flavor, and coffee flavor were derived as must have attributes (Figure 5b). In the MR environment, sweet taste, smooth swallowing, creamy flavor, milk flavor, and coffee flavor emerged as must have attributes (Figure 5c). When responses were analyzed irrespective of cognitive style, the MR context yielded a comparable or larger set of must-have attributes than the other contexts. Importantly, because the evaluation contexts were administered in a fixed sequence, cross-context differences should be interpreted as exploratory. In contrast, the must not have attributes included bitter taste and chalky coating in the real café environment; bitter taste, residual, chalky coating, and astringency in the sensory booth; and chalky coating in the MR environment.

#### 3.2.4. Ideal CATA Analysis by Evaluation Environment and Cognitive Style

**Figure 6:**
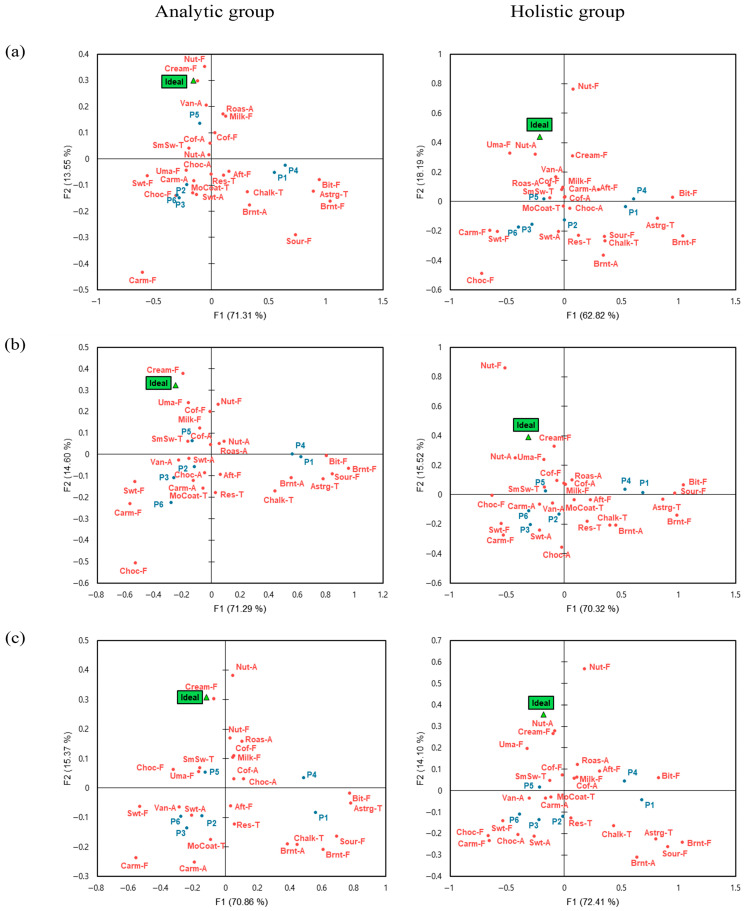
Representation of café latte samples, the ideal point, and sensory attributes in the first and second dimensions (F1 and F2) of correspondence analysis using the CATA method for different sensory evaluation environments and cognitive style. Sensory attribute abbreviations are defined in Table 2. (**a**): real café, (**b**): sensory booth, (**c**): mixed reality.

To address the primary objective, we examined cognitive style-related differences in ideal sensory profiles within each evaluation context by conducting Ideal CATA separately for analytic and holistic consumers in the real café, sensory booth, and MR contexts. The Ideal CATA data were mapped using CA within each context for each cognitive style group (Figure 6). The ideal product represents the benchmark of a perfect product as perceived by consumers, reflecting the essential attributes it should possess [15]. In the real environment, the analytic group explained 84.86% of the total variability, while the holistic group explained 81.01% (Figure 6a). The ideal product for the analytic group was closely associated with vanilla aroma, nutty flavor, and creamy flavor, whereas that for the holistic group was related to nutty aroma and umami. In the sensory booth, the analytic and holistic groups explained 85.89% and 85.84% of the variability, respectively (Figure 6b). The analytic group associated the ideal product with milk flavor, coffee flavor, umami and creamy flavor, while the holistic group associated it with nutty aroma, umami, and creamy flavor. In the MR environment, the analytic and holistic groups explained 85.93% and 86.51% of the variability, respectively (Figure 6c). The analytic group’s ideal product was linked to creamy flavor, whereas the holistic group’s ideal product was strongly associated with creamy flavor, umami, coffee flavor, and nutty aroma.

#### 3.2.5. RV Coefficients for Evaluation Environments and Cognitive Styles

To address the primary objective, RV coefficients were computed to compare the similarity of the CATA-derived product–attribute space between analytic and holistic consumers within each evaluation context, and additionally to compare similarity across contexts within each cognitive style group (Table 8). The RV coefficient is an index that quantifies the similarity between data matrices obtained under different conditions, with values closer to 1 indicating higher similarity [34]. Table 8 presents the RV coefficients calculated from CATA data according to cognitive style in the real café, sensory booth, and MR environments. For the analytic group, the RV coefficient between the real café and sensory booth was 0.980 (FDR-adjusted *p* < 0.05), between the real café and MR environment was 0.984 (FDR-adjusted *p* < 0.05), and between the sensory booth and MR was 0.996 (FDR-adjusted *p* < 0.05). For the holistic group, the RV coefficients were 0.968 (0.05 ≤ FDR-adjusted *p* < 0.10) between the real café and sensory booth, and 0.983 (0.05 ≤ FDR-adjusted *p* < 0.10) between the booth and MR. Although the RV coefficient of 0.988 between the real café and the MR environment was statistically significant based on the raw *p*-value (*p* < 0.05), it was not significant after FDR correction (FDR-adjusted *p* ≥ 0.10). All RV coefficients across environments were greater than 0.95, indicating very high similarity in CATA-based attributes. In the comparison between cognitive styles, RV coefficients between the analytic and holistic groups were also above 0.95 in the real café (0.959), sensory booth (0.994), and MR environment (0.983). Overall, the consistently high RV coefficients suggest that the CATA-derived product–attribute space was highly similar across evaluation contexts for both cognitive style groups.

### 3.3. RV Coefficients Between Descriptive Analysis and CATA

Table 9 presents the RV coefficients indicating the similarity between DA and CATA results across the three evaluation environments. The RV coefficient between DA and the real café was 0.884 (0.05 ≤ FDR-adjusted *p* < 0.10), and those between DA and the sensory booth and between DA and the MR environment were both 0.881 (0.05 ≤ FDR-adjusted *p* < 0.10), all showing statistically significant similarity. In addition, the RV coefficient between the real café and the MR environment was 0.986 (0.05 ≤ FDR-adjusted *p* < 0.10), between the real café and sensory booth was 0.982 (0.05 ≤ FDR-adjusted *p* < 0.10), and between the MR environment and sensory booth was 0.996 (0.05 ≤ FDR-adjusted *p* < 0.10). Overall, all comparisons yielded RV coefficients above 0.88, demonstrating a high level of similarity between DA and CATA results as well as among different evaluation environments.

## 4. Discussion

In consumer sensory research, it has been consistently emphasized that differences in evaluation environments play a critical role in shaping product acceptance. Accordingly, discussions have often centered on balancing sensory laboratories, which emphasize internal validity, with field tests, which highlight external validity. However, previous studies have largely been limited to examining the effects of evaluation environments—particularly immersive environments—without sufficiently accounting for the diverse factors that influence consumers’ sensory perception and preference. To address this gap, the present study examined cognitive style-related differences (analytic vs. holistic) in consumer liking, sensory perception, and ideal sensory profiles within each evaluation context, while additionally using mixed reality (MR) as a context-delivery tool to approximate a real consumption setting. To the best of our knowledge, this work provides one of the first integrated assessments combining MR-based context delivery with analytic–holistic cognitive style in consumer sensory evaluation.

### 4.1. Limited Influence of Cognitive Style in MR Contexts

This study classified consumers into analytic and holistic groups and compared their liking and sensory perception of café latte samples across three evaluation environments (real café, sensory booth, and MR café). The results showed that, in the MR environment, no significant differences in sensory perception were observed between the two cognitive style groups (Figure 4). In contrast, in the real café and sensory booth environments, the two groups demonstrated distinct sensory perceptions, suggesting that cognitive style-related differences were less pronounced under the MR evaluation context used in this study.

These factors can be understood as potential sources of psychological and cognitive bias in immersive environments. Such modulation may arise from several factors, including: (i) multidimensional stimuli in MR environments that may exceed users’ information-processing capacity, thereby inducing cognitive overload [35]; (ii) cybersickness arising from discrepancies between visual input and bodily sensations in virtual environments, which may cause physical discomfort and distort consumer responses [36]; (iii) novelty effects associated with the use of new technologies, which may temporarily amplify responses due to excitement or curiosity [37]; and (iv) physical pressure and discomfort from wearing head-mounted displays (HMD), which may act as confounding factors influencing overall sensory perception.

In the MR environment, consumers may have been more strongly influenced by environmental stimuli and device-related physical or psychological factors than by their inherent cognitive style when evaluating product characteristics. Therefore, the reduced differentiation between analytic and holistic consumers in the MR context should be interpreted as context dependent rather than as evidence that cognitive style is inherently less influential. This interpretation is consistent with previous studies reporting that immersive environments can distort user responses or weaken the effects of specific factors [35,36,37,38,39]. However, these MR-related variables (e.g., cognitive overload, cybersickness, novelty effects, and device discomfort) were not directly measured in the present study, which represents an important limitation.

Accordingly, strategies are needed to minimize these confounding factors in immersive sensory evaluation research. Recent studies have explored various methodological approaches. For instance, research employing Cave Automatic Virtual Environment systems has shown that participants can experience immersive visual and auditory stimuli within a real space while avoiding discomfort and cybersickness associated with HMD, thereby ensuring both ecological validity and experimental control [40]. In addition, immersive rooms and 360° video-based VR have been proposed as alternatives that maintain immersion while reducing technological unfamiliarity and cognitive overload [10]. Furthermore, recent studies have increasingly combined physiological measures such as EEG, heart rate, and eye-tracking to examine the multidimensional effects of immersive environments on consumers’ sensory and emotional responses [41]. Therefore, future studies incorporating such approaches are needed to more clearly disentangle cognitive style-related differences from context- and technology-induced effects.

### 4.2. Influence of Cognitive Style on Sensory Perception: Analytic Versus Holistic Groups

Based on the preceding discussion, cognitive style-related differences were less evident in the MR context used in this study; therefore, the present discussion primarily focuses on comparisons between the real café and sensory booth contexts, where cognitive style effects were more clearly observed. The results demonstrated clear differences in consumer responses to café latte samples according to cognitive style. Specifically, as illustrated in Figure 4, the analytic group tended to differentiate products based on individual attributes, whereas the holistic group appeared to integrate contextual cues with the overall product impression rather than relying on single attributes in isolation.

However, no significant differences in overall liking were observed between the two cognitive style groups. This suggests that product characteristics, particularly sucrose concentration, exerted a stronger influence on consumer liking than cognitive style (Table 4). Similarly, ref. [42] reported that sweetness is a primary determinant that enhances liking and consumption intent for foods. As highlighted by [6], the amplitude of inter-product differences can have a decisive impact on experimental comparisons; when the product space is broad, the effects of cognitive style may be diluted, preventing clear differences in liking from emerging. Accordingly, the absence of cognitive style effects on overall liking in the present study should be interpreted in light of the dominant role of sweetness rather than as evidence of a negligible role of cognitive style.

The comparison of RV coefficients derived from CATA data further revealed that the analytic group exhibited relatively higher similarity values with CATA frequency data than the holistic group (Table 8). This indicates that the analytic group maintained a stable evaluation structure across environments by focusing on single key attributes such as sucrose concentration. By contrast, although the holistic group also demonstrated high similarity, their reliance on contextual cues led to varying classification criteria across environments, resulting in lower consistency relative to the analytic group. These results are consistent with theoretical accounts describing analytic cognitive style as interpreting events in an independent and linear manner, while holistic cognitive style responds more sensitively to contextual cues and interprets events in interdependent and cyclical ways [43].

Previous studies [14,26,44] have also reported that holistic groups are more strongly influenced by consumption contexts than analytic groups, and the present findings further support this observation. Nevertheless, this study has several limitations. First, it focused on a single beverage category (café latte) with a limited set of samples and evaluation environments, which restricts the generalizability of the findings. In addition, the dominant role of sweetness in determining overall liking may have masked potential cognitive style-related differences. Second, cognitive style was operationalized by dichotomizing AHS scores, whereas AH theory conceptualizes cognitive style as a continuum. Models treating AHS as a continuous predictor (e.g., as a covariate in mixed-effects models) were not examined, which may limit the interpretation of cognitive style effects [14]. Finally, the relatively small sample size limited statistical power for group comparisons. Future studies should therefore include larger and more diverse consumer samples and examine cognitive style effects across a wider range of food categories and evaluation contexts.

### 4.3. Cognitive Style Differences in the Perception of the Ideal Product

Ideal product identification is a useful approach for understanding consumer liking by exploring the sensory characteristics associated with the ideal product [45]. While preference mapping relies on the existing product space, ideal product identification allows exploration of a broader sensory domain beyond the attributes represented by the tested products [45]. Accordingly, the present study employed the Ideal CATA approach to further examine how cognitive style may influence consumers’ perceptions of the ideal product.

The MR environment did not show clear cognitive style differences in the perception of the ideal product and exhibited patterns that differed from those observed in the real café and sensory booth contexts (Figure 6). In these two environments, the analytic group showed a broader range of sensory attributes associated with the ideal product than the holistic group, suggesting that analytic consumers may define the ideal product more explicitly as a combination of several key attributes and may respond relatively sensitively to the insufficiency or absence of these attributes [26]. In contrast, the holistic group showed a relatively narrower range of attributes associated with the ideal product and may be more likely to accept the coexistence of both preferred and less preferred characteristics within a single sample, judging ideality by integrating contextual information such as the overall situation and environment [26].

This tendency is broadly consistent with cognitive style theory. Analytic thinkers evaluate the fulfillment of individual attributes in a differentiated manner based on clear internal criteria, whereas holistic thinkers place greater weight on overall patterns and contextual meaning rather than on subtle differences between specific attributes [46]. Therefore, these findings suggest that the ideal product is not determined solely by the physical properties of the samples, but may vary depending on consumers’ cognitive styles and their context-dependent modes of interpretation. However, the present study focused on ideal product analysis as a preliminary stage of product optimization rather than conducting a full product optimization process.

### 4.4. Sensory Sensitivity in the MR Environments

As a secondary observation, one notable finding in this study was that, under the MR context, samples tended to be more clearly differentiated in terms of sensory responses and liking. In both the real café and MR environments, samples P3 and P5 received significantly higher liking scores than in the sensory booth (Table 5). Moreover, the MR context yielded a larger number of significant pairwise differences among samples in liking compared with the other two contexts (Table 5). These findings indicate that participants distinguished products more clearly in the MR context used in this study; however, these differences should be interpreted descriptively rather than causally. Similarly, in the CATA analysis, the MR environment produced the highest number of significantly discriminating attributes, with samples showing a clearer grouping based on specific sensory characteristics (Figure 3, Table 6). The penalty analysis also revealed that, in the MR environment, a broader range of attributes was identified as essential to the ideal product compared with the other two environments (Figure 6). In addition, CATA results obtained in the MR context showed high similarity with DA results (Table 9). Importantly, strong DA–CATA similarity was observed across all evaluation contexts, supporting previous findings that consumer-based CATA can provide reliable sensory information irrespective of context [47].

The enhanced sensory discrimination observed in the MR condition may be related to cross-modal interactions and environmental factors. The sensory booth represents a strictly controlled laboratory setting in which external cues are minimized and cross-modal interactions are relatively restricted [2]. By contrast, the real café reflects the natural context in which the product is actually consumed, whereas the MR environment can be regarded as a reconstruction of this real-world context within controlled laboratory conditions [2,48]. Cross-modal interaction occurs when stimulation in one sensory modality influences the perception of stimuli in another modality, for example when visual, auditory, or spatial cues modulate the perception of taste and flavor [49]. In a real café, background music, lighting, and spatial cues can affect the perception of coffee flavor [18], and the MR café, by recreating a similar café-like context and providing visual and auditory cues in a more tightly controlled manner, may have promoted cross-modal interactions, thereby contributing to clearer differentiation in sensory judgments.

In the broader literature, such effects are often discussed in relation to immersion and presence. Immersion generally describes an experimental setting in which users experience a sense of “being there” through the integration of multisensory cues such as visual, auditory, olfactory, and tactile stimuli [10]. Previous studies [50,51,52] suggest that higher levels of immersion enhance presence, which can alter evaluative processes such as attentional allocation and information processing, potentially increasing sensory sensitivity and discrimination. The influence of immersion and presence is also supported from a physiological perspective. MR environments may increase autonomic arousal and attentional focus, which in turn could modulate sensitivity to taste and flavor [53,54]. Studies conducted in immersive VR have shown that higher levels of presence and multisensory engagement are accompanied by changes in heart rate, skin conductance, and gaze patterns, which are associated with increased processing of sensory cues [54,55]. However, because immersion and presence were not directly measured, these interpretations remain speculative.

These findings also align with the conceptualization framework proposed by [56], which categorizes consumer perception into functional, emotional, and abstract dimensions. Functional conceptualization relates to associations with specific product attributes, emotional conceptualization concerns affective responses elicited by the product, and abstract conceptualization represents an intermediate stage linking functional and emotional judgments. From this perspective, in controlled environments such as sensory booths, consumers may tend to rely predominantly on functional judgments based on detailed sensory attributes and liking. In contrast, in uncontrolled environments such as real cafés, external stimuli may shift attentional focus, increasing the likelihood of decisions being driven by unconscious emotional responses. By comparison, the MR environment may have provided a context in which both functional and emotional judgments could operate simultaneously, enabling consumers to maintain functional evaluations of specific sensory attributes while also engaging affective components stimulated by the immersive experience. This interpretation is consistent with the findings of [5], which suggested that immersive environments can enhance both functional and emotional factors in consumer responses.

Overall, the findings of this study suggest that MR environments may provide a useful contextual framework for sensory evaluation, potentially facilitating more differentiated consumer responses under controlled conditions. Moreover, the use of the MR environment in this study also has several limitations. First, the level of immersion experienced by participants in the MR environment was not measured, which limits the interpretation of MR-related effects. Second, the evaluation was conducted in a single session, making it difficult to rule out potential novelty effects associated with short-term exposure.

Several methodological approaches have been proposed in previous studies to address these limitations. For instance, refs. [52,57,58] applied presence questionnaires to quantify immersion levels and incorporate them into the interpretation of consumer responses. Ref. [2] demonstrated that the effects of immersion can vary depending on product characteristics by verifying differences in context sensitivity. Refs. [59,60] further confirmed that repeated and long-term exposure to immersive environments can mitigate initial novelty effects, leading to more stable consumer responses over time.

## 5. Conclusions

This study examined how consumer liking, sensory perception, and ideal product perception vary according to cognitive style across three evaluation contexts (real café, sensory booth, and mixed reality). The primary contribution of this work lies in empirically demonstrating that sensory perception and ideal sensory profiles can differ between analytic and holistic consumers within each context. In addition, the findings suggest that mixed reality (MR) has the potential to serve as a context-delivery approach capable of eliciting more differentiated consumer responses under controlled conditions. However, differences observed across contexts should not be interpreted causally.

Several limitations should be considered when interpreting these findings. Participants were recruited from a single university population, and the relatively small group sizes after classification by cognitive style may have reduced statistical power to detect subtle effects. In addition, the fixed order of context presentation and the dichotomization of Analysis–Holism Scale scores limit the interpretation of context effects. Future research should include more diverse participant samples, apply randomized or counterbalanced context orders, and incorporate additional measures related to immersion and sensory sensitivity.

Overall, this study supports the importance of considering cognitive style in consumer sensory research and highlights the potential of MR-based context delivery as a complementary methodological approach under controlled evaluation conditions.

## Figures and Tables

**Table 1:** Composition of café latte samples with different contents of sugar, espresso and milk.

Product	Sugar (% *w*/*w*)	Espresso (% *w*/*w*)	Milk (% *w*/*w*)
P1	0	33.3	66.7
P2	2.5	32.5	65.0
P3	5	31.7	63.3
P4	0	25.0	75.0
P5	2.5	24.4	73.1
P6	5	23.8	71.3

**Table 2:** Developed a lexicon for the café latte sample.

	Attribute	Definition	Reference [Intensity]
Aroma	Coffee (Cof-A) ^(1)^	Intensity of the coffee aroma of cafe latte	Nespresso Capriccio [9] (Nestlé Nespresso S.A., Lausanne, Switzerland)
	Chocolate (Choc-A)	Intensity of the chocolate aroma of cafe latte	Excellence 85% cocoa dark chocolate bar [13] (Lindt & Sprüngli AG, Kilchberg, Switzerland)
	Nutty (Nut-A)	Intensity of the nutty aroma of cafe latte	Roasted almond [8]
	Caramelized (Carm-A)	Intensity of the caramelized aroma of cafe latte	50% caramel syrup solution [8] (1883 Masion Routin, La Motte-Servolex, France)
	Roasted (Roas-A)	Intensity of the roasted aroma of cafe latte	Nespresso Ispirazione Napoli [11] (Nestlé Nespresso S.A., Lausanne, Switzerland)
	Vanilla (Van-A)	Intensity of the vanilla aroma of cafe latte	Nespresso Vanilla Éclair [9] (Nestlé Nespresso S.A., Lausanne, Switzerland)
	Sweet (Swt-A)	Intensity of the sweet aroma of cafe latte	Granola cereal [7] (Kellogg’s, Battle Creek, MI, USA)
	Burnt (Brnt-A)	Intensity of the burnt aroma of cafe latte	Roasted almond [6]
Taste/ Flavor	Sweet (Swt-F)	Intensity of the sweet taste of cafe latte	2%, 5% sucrose solution [2,5]
	Sour (Sour-F)	Intensity of the sour taste of cafe latte	0.05% citric acid solution [2]
	Bitter (Bit-F)	Intensity of the bitter taste of cafe latte	Nespresso Ispirazione Napoli [9] (Nestlé Nespresso S.A., Lausanne, Switzerland)
	Umami (Uma-F)	Intensity of the umami taste of cafe latte	0.02% MSG solution [2]
	Burnt (Brnt-F)	Intensity of the burnt flavor of cafe latte	Roasted almond [8]
	Coffee (Cof-F)	Intensity of the coffee flavor of cafe latte	Americano 40 mL [8]/(200 mL Nespresso Ispirazione Napoli Espresso and 200 mL water)
	Caramelized (Carm-F)	Intensity of the caramelized flavor of cafe latte	5% caramel syrup solution [3] (1883 Masion Routin, La Motte-Servolex, France)
	Nutty (Nut-F)	Intensity of the nutty flavor of cafe latte	Roasted almond [6]
	Chocolate (Choc-F)	Intensity of the chocolate flavor of cafe latte	Excellence 85% cocoa dark chocolate bar [9] (Lindt & Sprüngli AG, Kilchberg, Switzerland)
	Milk (Milk-F)	Intensity of the milk flavor of cafe latte	Whole milk [10] (Seoul Milk Cooperative, Seoul, Republic of Korea)
	Creamy (Cream-F)	Intensity of the savory flavor of milk of cafe latte	Whipped cream [10] (Maeil Dairy Industry Co., Ltd., Seoul, Republic of Korea)
	Aftertaste (Aft-F)	Intensity of the aftertaste of cafe latte	Yogurt [7] (Binggrae Co., Ltd., Seoul, Republic of Korea), Greek yogurt [12] (Seoul Milk Cooperative, Seoul, Republic of Korea)
Texture	Smooth swallowing (SmSw-T)	Intensity of the smooth swallowing of milk of cafe latte	Water [2]; Yogurt [8] (Binggrae Co., Ltd., Seoul, Republic of Korea)
	Mouth coating (MoCoat-T)	Intensity of the mouth coating of milk of cafe latte	Whole milk [5] (Seoul Milk Cooperative, Seoul, Republic of Korea)
	Residual (Res-T)	Intensity of the aroma or flavor remaining in the mouth after tasting cafe latte	Yogurt [8] (Binggrae Co., Ltd., Seoul, Republic of Korea)
	Chalky coating (Chalk-T)	Intensity of the chalky coating of milk of cafe latte	Yogurt [7] (Binggrae Co., Ltd., Seoul, Republic of Korea)
	Astringency (Astrg-T)	Intensity of the astringency of cafe latte	50% black tea solution [2], black tea (Dong-suh Food Co., Ltd., Seoul, Republic of Korea)

^(1)^ Sensory attribute abbreviations are indicated in parentheses: aroma (-A), taste/flavor (-F), and texture (-T).

**Table 3:** Mean and standard deviations for the sensory attributes obtained from descriptive analysis for the café latte samples.

Attribute	P1	P2	P3	P4	P5	P6	*p*-Value	Partial η^2^
**Aroma**								
Coffee	6.8 ± 0.5 ^(1) a (2)^	6.7 ± 0.9 ^a^	7.1 ± 1.3 ^a^	6.9 ± 0.4 ^a^	6.4 ± 0.5 ^a^	5.4 ± 0.9 ^b^	0.003	0.336
Chocolate	6.3 ± 1.6 ^a^	6.1 ± 1.0 ^a^	6.6 ± 1.4 ^a^	5.9 ± 1.4 ^a^	5.8 ± 1.2 ^a^	4.9 ± 1.3 ^a^	0.236	0.145
Nutty	4.8 ± 0.5 ^a^	4.6 ± 0.7 ^a^	5.1 ± 1.1 ^a^	5.3 ± 0.9 ^a^	4.6 ± 0.9 ^a^	4.1 ± 1.3 ^a^	0.168	0.164
Caramelized	3.8 ± 0.7 ^c^	4.4 ± 0.7 ^bc^	5.3 ± 0.7 ^ab^	4.6 ± 1.1 ^abc^	4.8 ± 1.1 ^abc^	5.6 ± 1.3 ^a^	0.010	0.294
Roasted	7.2 ± 0.7 ^a^	7.6 ± 0.8 ^a^	7.4 ± 0.6 ^a^	7.6 ± 0.5 ^a^	6.7 ± 0.5 ^a^	6.0 ± 1.5 ^a^	0.016	0.274
Vanilla	4.7 ± 1.1 ^bc^	5.6 ± 0.6 ^ab^	6.2 ± 0.6 ^a^	4.1 ± 1.4 ^c^	5.6 ± 1.2 ^ab^	5.6 ± 0.8 ^ab^	0.002	0.350
Sweet	3.4 ± 0.8 ^b^	4.6 ± 1.0 ^ab^	5.4 ± 1.0 ^a^	3.5 ± 0.9 ^b^	5.7 ± 1.3 ^a^	5.8 ± 1.6 ^a^	<0.0001	0.450
Burnt	5.9 ± 0.8 ^a^	5.5 ± 0.7 ^ab^	4.6 ± 0.8 ^bc^	6.0 ± 1.0 ^a^	4.8 ± 0.7 ^bc^	3.9 ± 1.2 ^c^	0.000	0.442
**Taste/Flavor**								
Sweet	1.6 ± 0.7 ^c^	3.9 ± 0.6 ^b^	5.9 ± 0.9 ^a^	1.6 ± 0.8 ^c^	3.8 ± 0.5 ^b^	6.6 ± 1.5 ^a^	<0.0001	0.839
Sour	1.9 ± 0.6 ^a^	1.1 ± 0.2 ^bc^	0.8 ± 0.3 ^c^	1.8 ± 0.5 ^a^	1.3 ± 0.5 ^b^	0.9 ± 1.5 ^bc^	<0.0001	0.529
Bitter	7.8 ± 0.7 ^a^	6.2 ± 0.6 ^b^	4.5 ± 1.0 ^c^	7.4 ± 1.1 ^a^	5.8 ± 1.5 ^b^	3.9 ± 1.6 ^c^	<0.0001	0.643
Umami	2.0 ± 0.7 ^a^	2.4 ± 0.8 ^a^	2.9 ± 11.4 ^a^	1.7 ± 0.5 ^a^	2.1 ± 0.5 ^a^	2.8 ± 1.1 ^a^	0.070	0.209
Burnt	5.9 ± 1.0 ^a^	5.3 ± 0.5 ^ab^	4.1 ± 0.4 ^bc^	5.9 ± 1.1 ^a^	4.8 ± 0.7 ^bc^	3.4 ± 0.8 ^d^	<0.0001	0.619
Coffee	6.8 ± 0.8 ^a^	5.4 ± 0.9 ^b^	5.2 ± 1.3 ^bc^	5.9 ± 0.7 ^ab^	5.3 ± 1.2 ^b^	4.1 ± 1.4 ^c^	0.001	0.380
Caramelized	2.7 ± 0.7 ^d^	4.7 ± 0.8 ^bc^	5.9 ± 1.7 ^ab^	3.8 ± 1.7 ^cd^	6.6 ± 1.8 ^a^	7.5 ± 2.3 ^a^	<0.0001	0.546
Nutty	6.0 ± 1.5 ^a^	5.3 ± 1.3 ^a^	4.6 ± 1.8 ^a^	6.8 ± 1.6 ^a^	5.9 ± 1.4 ^a^	5.6 ± 1.9 ^a^	0.177	0.161
Chocolate	5.3 ± 1.5 ^a^	5.6 ± 1.1 ^a^	5.2 ± 1.4 ^a^	5.2 ± 1.4 ^a^	4.7 ± 1.0 ^a^	5.2 ± 2.0 ^a^	0.902	0.036
Milk	6.6 ± 1.0 ^ab^	6.7 ± 1.0 ^ab^	5.6 ± 1.3 ^b^	7.6 ± 1.3 ^a^	6.8 ± 0.9 ^ab^	6.5 ± 1.3 ^ab^	0.039	0.236
Creamy	6.6 ± 1.5 ^ab^	6.1 ± 1.0 ^ab^	5.3 ± 1.2 ^b^	7.5 ± 1.7 ^a^	6.6 ± 1.3 ^ab^	5.6 ± 1.5 ^b^	0.040	0.236
Aftertaste	6.3 ± 1.0 ^a^	7.1 ± 1.2 ^a^	7.4 ± 1.1 ^a^	6.7 ± 1.2 ^a^	7.3 ± 0.9 ^a^	7.6 ± 1.1 ^a^	0.216	0.150
**Texture**								
Smooth swallowing	5.3 ± 1.0 ^bc^	6.4 ± 0.9 ^ab^	7.4 ± 0.9 ^a^	4.8 ± 1.5 ^c^	6.9 ± 1.5 ^a^	7.8 ± 1.4 ^a^	<0.0001	0.458
Mouth coating	4.4 ± 1.3 ^b^	5.4 ± 1.2 ^ab^	6.2 ± 1.0 ^a^	4.1 ± 1.3 ^b^	5.1 ± 1.1 ^ab^	6.1 ± 1.5 ^a^	0.006	0.316
Residual	5.9 ± 0.8 ^b^	6.9 ± 0.9 ^ab^	7.5 ± 11.3 ^a^	5.9 ± 1.1 ^b^	6.9 ± 1.0 ^ab^	7.2 ± 1.0 ^a^	0.013	0.283
Chalky coating	6.7 ± 2.2 ^a^	6.6 ± 1.6 ^a^	6.4 ± 1.2 ^a^	7.0 ± 1.8 ^a^	6.6 ± 1.7 ^a^	6.5 ± 1.4 ^a^	0.988	0.014
Astringency	5.4 ± 2.6 ^a^	4.8 ± 2.1 ^ab^	2.8 ± 2.0 ^bc^	5.1 ± 2.2 ^a^	3.9 ± 1.6 ^abc^	2.5 ± 1.2 ^c^	0.020	0.265

^(1)^ Mean ± standard deviation. ^(2)^ Different superscripts in the same column for each descriptor indicate significant differences at *p* < 0.05.

**Table 4:** Overall liking scores of six cafe latte samples evaluated under different sensory environments and cognitive styles.

Overall Liking ^(1)^						
	Real Café		Sensory Booth		Mixed Reality	
Sample	Analytic ^(4)^	Holistic	Analytic	Holistic	Analytic	Holistic
P1	4.8 ^b (2)^_A__(3)_	5.6 ^b^_A_	4.6 ^b^ _A_	4.7 ^b^_A_	5.0 ^b^ _A_	4.9 ^b^_A_
P2	6.7 ^a^ _A_	6.3 ^ab^_A_	6.1 ^a^ _A_	5.7 ^a^ _A_	6.3 ^a^_A_	5.9 ^a^_A_
P3	7.2 ^a^ _A_	6.7 ^a^_A_	6.5 ^a^ _A_	6.1 ^a^_A_	6.8 ^a^_A_	6.3 ^a^_A_
P4	3.8 ^c^ _A_	3.9 ^c^_A_	4.3 ^b^ _A_	3.9 ^a^_A_	3.9 ^c^_A_	4.0 ^c^_A_
P5	6.5 ^a^ _A_	6.5 ^a^_A_	6.1 ^a^ _A_	5.9 ^a^_A_	6.6 ^a^_A_	6.4 ^a^_A_
P6	6.6 ^a^ _A_	6.3 ^ab^_A_	6.3 ^a^ _A_	6.4 ^a^_A_	6.8 ^a^ _A_	6.8 ^a^_A_

^(1)^ Overall liking test was evaluated using 9 points (1-dislike extremely, 9-like extremely). ^(2)^ Means with the different superscripts in the same column for the samples are significantly different at *p* < 0.05. ^(3)^ Subscript letters indicate comparisons between cognitive styles within rows; no significant differences were observed (*p* > 0.05). ^(4)^ Analytic group: N = 34; Holistic group: N = 43.

**Table 5:** Overall liking scores of six café latte samples evaluated under different sensory environments.

Overall Liking ^(1)^			
Sample	Real Café	Sensory Booth	Mixed Reality
P1	5.2 ^b (2)^_A (3)_	4.6 ^b^ _A_	5.0 ^c^ _A_
P2	6.6 ^a^ _A_	5.9 ^a^ _B_	6.1 ^b^ _B_
P3	7.0 ^a^ _A_	6.3 ^a^ _B_	6.6 ^ab^ _AB_
P4	3.9 ^c^ _A_	4.1 ^b^ _A_	3.9 ^d^ _A_
P5	6.5 ^a^ _A_	6.0 ^a^ _B_	6.5 ^ab^ _A_
P6	6.5 ^a^ _A_	6.4 ^a^ _A_	6.8 ^a^ _A_

^(1)^ Overall liking test was evaluated using 9 points (1-dislike extremely, 9-like extremely). ^(2)^ Means with the different superscripts in the same column for the samples are significantly different at *p* < 0.05. ^(3)^ Means with the different subscripts in the same row for the environments are significantly different at *p* < 0.05.

**Table 6:** Frequencies of the sensory attributes selected using the check-all-that-apply (CATA) method for cafe latte samples according to different sensory evaluation environments.

	Real Café						Sensory Booth						Mixed Reality					
	P1	P2	P3	P4	P5	P6	P1	P2	P3	P4	P5	P6	P1	P2	P3	P4	P5	P6
**Aroma**																		
Coffee	61 a ^(1)^	61 a	60 a	53 a	65 a	59 a	63 a	56 a	62 a	52 a	62 a	63 a	68 a	63 a	65 a	59 a	60 a	60 a
Chocolate	14 a	15 a	11 a	7 a	9 a	9 a	11 a	13 a	15 a	9 a	10 a	14 a	8 a	13 a	13 a	9 a	11 a	10 a
Nutty	5 a	11 a	10 a	13 a	13 a	15 a	5 a	7 a	9 a	13 a	10 a	12 a	11 a	8 a	14 a	11 a	15 a	9 a
Caramelized	20 a	22 a	23 a	14 a	19 a	24 a	16 bc	32 a	26 abc	14 c	28 abc	30 ab	20 bc	23 abc	38 a	12 c	17 c	33 ab
Roasted	28 ab	19 b	34 ab	31 ab	37 b	30 ab	44 a	30 a	36 a	37 a	39 a	40 a	43 a	29 a	29 a	38 a	37 a	41 a
Vanilla	16 a	16 a	18 a	16 a	21 a	20 a	12 a	20 a	19 a	10 a	16 a	20 a	8 b	18 ab	18 ab	8 b	23 a	26 a
Sweet	21 b	31 ab	35 ab	23 ab	25 ab	38 a	19 bc	37 a	39 a	16 c	33 ab	30 abc	14 c	40 a	45 a	22 bc	37 ab	42 a
Burnt	26 a	14 ab	11 b	15 ab	14 ab	14 ab	23 a	11 b	11 b	15 ab	10 b	7 b	28 a	16 ab	11 b	17 ab	11 b	10 b
**Taste/Flavor**																		
Sweet	4 b	72 a	75 a	0 b	74 a	77 a	1 b	70 a	73 a	5 b	64 a	72 a	4 b	69 a	73 a	3 b	70 a	77 a
Sour	11 ab	7 ab	5 ab	13 ab	6 ab	3 b	14 ab	5 bc	2 c	20 ab	7 bc	3 c	9 ab	6 ab	3 b	11 a	2 b	2 b
Bitter	54 a	16 b	6 b	46 a	8 b	4 b	63 a	25 c	6 d	44 b	12 cd	5 d	54 a	15 c	11 c	34 b	11 c	6 c
Umami	5 b	9 ab	16 ab	6 b	15 ab	18 a	7 a	11 a	12 a	11 a	16 a	12 a	8 b	13 ab	21 ab	13 ab	20 a	25 a
Burnt	34 a	8 b	7 b	25 a	5 b	1 b	37 a	11 bc	5 c	24 ab	6 c	5 c	40 a	16 bc	13 bc	22 b	20 b	3 c
Coffee	50 a	43 ab	43 ab	35 b	44 ab	42 ab	47 a	47 a	44 a	34 a	46 a	41 a	55 a	55 a	50 a	44 a	58 a	53 a
Caramelized	5 bc	20 a	42 bc	0 c	15 bc	44 a	2 d	15 cd	31 ab	3 d	19 bc	35 bc	1 c	23 b	43 a	2 c	23 bc	43 a
Nutty	4 b	6 ab	8 ab	13 ab	14 a	5 ab	6 b	5 b	6 b	7 b	18 a	5 b	13 a	11 a	8 a	11 a	16 a	8 a
Chocolate	5 b	13 ab	11 ab	2 b	9 b	22 a	2 c	9 bc	18 ab	1 c	10 bc	26 a	3 c	10 bc	16 ab	3 c	14 ab	22 a
Milk	39 a	34 a	30 a	40 a	45 a	35 a	32 a	41 a	40 a	36 a	43 a	33 a	36 ab	48 a	31 b	46 ab	46 ab	37 ab
Creamy	35 ab	31 ab	25 b	24 b	42 a	27 ab	22 a	32 a	28 a	28 a	34 a	28 a	23 b	30 ab	38 ab	31 ab	42 a	29 ab
Aftertaste	20 a	10 a	14 a	22 a	17 a	16 a	14 a	11 a	13 a	13 a	11 a	13 a	23 a	16 a	21 a	21 a	17 a	19 a
**Texture**																		
Smooth swallowing	48 b	64 a	66 a	30 c	60 ab	61 ab	37 b	59 a	59 a	38 b	55 a	66 a	42 b	60 a	63 a	35 b	58 a	69 a
Mouth coating	21 a	25 a	26 a	19 a	25 a	29 a	20 a	32 a	25 a	25 a	28 a	26 a	22 ab	29 ab	29 ab	20 b	33 ab	36 a
Residual	29 a	26 a	24 a	28 a	24 a	31 a	31 a	23 a	31 a	29 a	26 a	34 a	36 a	33 a	34 a	28 a	38 a	37 a
Chalky coating	24 ab	25 ab	18 b	40 a	28 ab	18 b	32 ab	24 b	20 b	41 a	21 b	19 b	34 ab	22 bc	19 bc	42 a	18 c	22 bc
Astringency	25 b	10 c	8 c	41 a	14 bc	4 c	34 a	10 b	7 b	32 a	12 b	8 b	34 a	11 b	12 b	31 a	11 b	5 b

^(1)^ Frequencies with the same letter within the same row are not significantly different at q < 0.05 (FDR-adjusted *p*-values).

**Table 7:** Relationships between the attributes and samples based on multiple discriminant analysis using the CATA method for different sensory evaluation environments.

	Real Café						Sensory Booth						Mixed Reality					
	P1	P2	P3	P4	P5	P6	P1	P2	P3	P4	P5	P6	P1	P2	P3	P4	P5	P6
**Aroma**																		
Coffee	+0.419	+0.184	−0.406	+0.145	+0.167	−0.590	+0.508	−0.699	−0.255	+0.199	−0.279	−0.184	+0.950 ^(1)^	−0.517	−0.745 ^(2)^	+0.877 ^(1)^	−0.831 ^(2)^	−0.949 ^(2)^
Chocolate	+0.355	+0.492	−0.090	−0.154	−0.370	−0.189	−0.438	+0.395	+0.716 ^(1)^	−0.643	−0.148	+0.659	−0.504	+0.737 ^(1)^	+0.474	−0.339	+0.306	+0.179
Nutty	−0.612	+0.064	+0.172	−0.037	+0.405	+0.382	−0.244	−0.385	+0.002	+0.247	+0.136	+0.195	+0.175	−0.509	−0.086	+0.282	+0.164	−0.357
Caramelized	−0.420	+0.660	+0.737 ^(1)^	−0.827 ^(2)^	−0.103	+0.745 ^(1)^	−0.854 ^(2)^	+0.841 ^(1)^	+0.790 ^(1)^	−0.900 ^(2)^	+0.646	+0.736 ^(1)^	−0.474	+0.195	+0.855 ^(1)^	−0.676	−0.024	+0.655
Roasted	−0.009	−0.725 ^(2)^	−0.012	0.297	+0.270	−0.163	+0.728 ^(1)^	−0.942 ^(2)^	−0.633	+0.645	−0.455	−0.455	+0.732 ^(1)^	−0.781 ^(2)^	−0.821 ^(2)^	+0.760 ^(1)^	−0.481	−0.552
Vanilla	−0.581	−0.135	+0.308	−0.262	+0.736 ^(1)^	+0.426	−0.824 ^(2)^	+0.789 ^(1)^	+0.888 ^(1)^	−0.936 ^(2)^	+0.416	+0.831 ^(1)^	−0.907 ^(2)^	+0.563	+0.653	−0.870 ^(2)^	+0.847 ^(1)^	+0.921 ^(1)^
Sweet	−0.738 ^(2)^	+0.504	+0.819 ^(1)^	−0.666	+0.021	+0.866 ^(1)^	−0.820 ^(2)^	+0.817 ^(1)^	+0.869 ^(1)^	−0.895 ^(2)^	+0.637	+0.632	−0.966 ^(2)^	+0.712 ^(1)^	+0.861 ^(1)^	−0.819 ^(2)^	+0.663	+0.834 ^(1)^
Burnt	+0.941 ^(1)^	−0.458	−0.750 ^(2)^	+0.556	−0.623	−0.672	+0.979 ^(1)^	−0.544	−0.846 ^(2)^	+0.792 ^(1)^	−0.712 ^(2)^	−0.870 ^(2)^	+0.980 ^(1)^	−0.477	−0.810 ^(2)^	+0.782 ^(1)^	−0.781 ^(2)^	−0.888 ^(2)^
**Taste/Flavor**																		
Sweet	−0.932 ^(2)^	+0.787 ^(1)^	+0.872 ^(1)^	−0.931 ^(2)^	+0.667	+0.856 ^(1)^	−0.957 ^(2)^	+0.732 ^(1)^	+0.940	−0.955 ^(2)^	+0.705	+0.846 ^(1)^	−0.943 ^(2)^	+0.750 ^(1)^	+0.835 ^(1)^	−0.960 ^(2)^	+0.778 ^(1)^	+0.884 ^(1)^
Sour	+0.843 ^(1)^	−0.622	−0.889 ^(2)^	+0.964 ^(1)^	−0.535	−0.945 ^(2)^	+0.808 ^(1)^	−0.658	−0.929 ^(2)^	+0.995 ^(1)^	−0.537	−0.837 ^(2)^	+0.831 ^(1)^	−0.474	−0.810 ^(2)^	+0.959 ^(1)^	−0.770 ^(2)^	−0.874 ^(2)^
Bitter	+0.969 ^(1)^	−0.652	−0.892 ^(2)^	+0.893 ^(1)^	−0.695	−0.888 ^(2)^	+0.986 ^(1)^	−0.493	−0.947 ^(2)^	+0.881 ^(1)^	−0.744 ^(2)^	−0.906 ^(2)^	+0.999 ^(1)^	−0.649	−0.823 ^(2)^	+0.870 ^(1)^	−0.793 ^(2)^	−0.913 ^(2)^
Umami	−0.898 ^(2)^	+0.337	+0.878 ^(1)^	−0.781	+0.582	+0.900 ^(1)^	−0.652	+0.136	+0.374	−0.293	+0.886 ^(1)^	+0.323	−0.846 ^(2)^	+0.135	+0.708 ^(1)^	−0.635	+0.627	+0.873 ^(1)^
Burnt	+0.984 ^(1)^	−0.666	−0.829 ^(2)^	+0.845 ^(1)^	−0.703	−0.883 ^(2)^	+0.998_1)_	−0.579	−0.918 ^(2)^	+0.866 ^(1)^	−0.781 ^(2)^	−0.857 ^(2)^	+0.982 ^(1)^	−0.526	−0.742 ^(2)^	+0.790 ^(1)^	−0.792 ^(2)^	−0.923 ^(2)^
Coffee	+0.665	−0.026	−0.304	+0.057	−0.401	−0.392	+0.368	+0.254	−0.202	−0.069	−0.040	−0.404	+0.580	−0.023	−0.755 ^(2)^	+0.386	−0.039	−0.614
Caramelized	−0.768 ^(2)^	+0.470	+0.953 ^(1)^	−0.845 ^(2)^	+0.184	+0.945 ^(1)^	−0.875 ^(2)^	+0.337	+0.958 ^(1)^	−0.878 ^(2)^	+0.494	+0.983 ^(1)^	−0.889 ^(2)^	+0.469	+0.960 ^(1)^	−0.910 ^(2)^	+0.523	+0.944 ^(1)^
Nutty	−0.093	−0.382	−0.278	+0.472	+0.486	−0.392	−0.131	−0.139	−0.078	+0.013	+0.739 ^(1)^	−0.162	+0.512	−0.218	−0.756 ^(2)^	+0.536	+0.105	−0.678
Chocolate	−0.714 ^(2)^	+0.623	+0.713 ^(1)^	−0.825 ^(2)^	+0.208	+0.949 ^(1)^	−0.791 ^(2)^	+0.268	+0.886 ^(1)^	−0.828 ^(2)^	+0.357	+0.990 ^(1)^	−0.891 ^(2)^	+0.391	+0.828 ^(1)^	−0.891 ^(2)^	+0.642	+0.991 ^(1)^
Milk	+0.521	−0.558	−0.851 ^(2)^	+0.754 ^(1)^	+0.114	−0.743 ^(2)^	−0.228	+0.343	+0.004	+0.048	+0.552	−0.282	+0.277	+0.088	−0.716 ^(2)^	+0.606	+0.001	−0.522
Creamy	+0.261	+0.053	−0.409	+0.057	+0.460	−0.393	−0.468	+0.395	+0.106	−0.079	+0.695	+0.010	−0.385	+0.060	+0.191	−0.042	+0.596	+0.038
Aftertaste	+0.697	−0.958 ^(2)^	−0.740 ^(2)^	+0.891 ^(1)^	−0.413	−0.688	+0.767 ^(1)^	−0.820 ^(2)^	−0.607	+0.744 ^(1)^	−0.828 ^(2)^	−0.473	+0.846 ^(1)^	−0.861 ^(2)^	−0.583	+0.857 ^(1)^	−0.843 ^(2)^	−0.741 ^(2)^
**Texture**																		
Smooth swallowing	−0.668	+0.843 ^(1)^	+0.827 ^(1)^	−0.952 ^(2)^	+0.414	+0.721 ^(1)^	−0.945 ^(2)^	+0.635	+0.921 ^(1)^	−0.908 ^(2)^	+0.562	+0.938 ^(1)^	−0.887 ^(2)^	+0.697	+0.831 ^(1)^	−0.969 ^(2)^	+0.624	+0.938 ^(1)^
Mouth coating	−0.847 ^(2)^	+0.671	+0.867 ^(1)^	−0.893 ^(2)^	+0.317	+0.986 ^(1)^	−0.444	+0.680	+0.128	−0.138	+0.411	+0.050	−0.848 ^(2)^	+0.525	+0.552	−0.818 ^(2)^	+0.829 ^(1)^	+0.890 ^(1)^
Residual	+0.582	−0.469	−0.590	+0.593	−0.781 ^(1)^	−0.294	+0.399	−0.848 ^(2)^	−0.227	+0.399	−0.585	+0.003	+0.490	−0.385	−0.540	+0.200	+0.023	−0.327
Chalky coating	+0.475	−0.519	−0.789 ^(2)^	+0.899 ^(1)^	−0.151	−0.777 ^(2)^	+0.788 ^(1)^	−0.577	−0.886 ^(2)^	+0.983 ^(1)^	−0.628	−0.816 ^(2)^	+0.786 ^(1)^	−0.640	−0.804 ^(2)^	+0.975 ^(1)^	−0.732	−0.779 ^(2)^
Astringency	+0.737 ^(1)^	−0.747 ^(2)^	−0.856 ^(2)^	+0.997 ^(1)^	−0.437	−0.881 ^(2)^	+0.948 ^(1)^	−0.705	−0.956 ^(2)^	+0.971 ^(1)^	−0.683	−0.868 ^(2)^	+0.945 ^(1)^	−0.701	−0.818 ^(2)^	+0.962 ^(1)^	−0.762	−0.935 ^(2)^

^(1)^ Strong positive correlations are defined as coefficients ≥ 0.707. ^(2)^ Strong negative correlations are defined as coefficients ≤ −0.707.

**Table 8:** Comparison of RV coefficients between real café and different evaluation environments according to cognitive style using the CATA method.

	Real Café A ^(1)^	Sensory Booth A	Mixed Reality A	Real Café H	Sensory Booth H	Mixed Reality H
Real café A		0.980 ** ^(2)^	0.984 **	0.959 **	0.971 **	0.952 **
Sensory booth A			0.996 **	0.974 **	0.994 *	0.984 *
Mixed reality A				0.973 **	0.993 *	0.983 *
Real café H					0.968 *	0.988 ^NS^
Sensory booth H						0.983 *
Mixed reality H						

^(1)^ A = Analytic group, H = Holistic group. ^(2)^ ** FDR-adjusted *p* < 0.05; * 0.05 ≤ FDR-adjusted *p* < 0.10; NS = not significant.

**Table 9:** Comparison of RV coefficients between descriptive analysis and the CATA method was conducted for different sensory evaluation environments.

	DA	Real Café	Sensory Booth	MR
DA	1.000	0.884 ** ^(1)^	0.881 **	0.881 **
Real café		1.000	0.986 **	0.982 **
Sensory booth			1.000	0.996 *
MR				

^(1)^ ** FDR-adjusted *p* < 0.05, * 0.05 ≤ FDR-adjusted *p* < 0.10.

## Data Availability

The data presented in this study are available on request from the corresponding author. The data are not publicly available due to privacy restrictions.

## Abbreviations

The following abbreviations are used in this manuscript:

XR	Extended reality
VR	Virtual reality
AR	Augmented reality
MR	Mixed reality
AHS	Analytic-Holistic Scale
CATA	Check-All-That-Apply
DA	Descriptive analysis
